# Association between Telomere Length and Pediatric Obesity: A Systematic Review

**DOI:** 10.3390/nu14061244

**Published:** 2022-03-15

**Authors:** Christina Raftopoulou, George Paltoglou, Evangelia Charmandari

**Affiliations:** 1 Division of Endocrinology and Metabolism, Center of Clinical, Experimental Surgery and Translational Research, Biomedical Research Foundation of the Academy of Athens, 11527 Athens, Greece; xraftopoulou@yahoo.gr; 2 Division of Endocrinology, Metabolism and Diabetes, First Department of Pediatrics, National and Kapodistrian University of Athens Medical School, Aghia Sophia Children’s Hospital, 11527 Athens, Greece; gpaltoglou@med.uoa.gr

**Keywords:** obesity, overweight, leukocyte telomere length, childhood, adolescence

## Abstract

Objective: Telomere length (TL) is a robust marker of biological aging, and increased telomere attrition is noted in adults with obesity. The primary objective of this systematic review was to summarize current knowledge on the effects of childhood obesity in TL. The secondary objective was to assess the effect of weight management interventions in TL. Methods: The following databases were searched: PubMed, Scopus, Web of Science and Heal-link.gr from inception to September 2021. The search was performed using the following combinations of terms: “telomer*” [All Fields] AND (“length” [All Fields] OR “lengths” [All Fields]) AND “obes*” [All Fields] AND (“child*” [All Fields] OR “adolescen*” [All Fields]). Results: A total of 16 original articles were included in this systematic review. Eleven of them were cross-sectional and five were lifestyle interventions. Conclusions: There was a tendency towards a negative association between childhood obesity and TL. Life-style interventions in children have been associated with increased TL peripherally, indicating a possible association of the redistribution of younger cells in the periphery with the favorable effect of these interventions. Further prospective studies with larger sample sizes that employ other markers of cell aging would potentially elucidate this important mechanistic relation.

## 1. Introduction

Telomeres in humans are the natural end of chromosomes and consist of highly conserved hexameric (5′-TTAGGG-3′) tandem repeat DNA sequences and proteins, that cap and protect eukaryotic chromosome ends from being processed as DNA double-strand breaks (DSBs) [1]. Telomeres shorten progressively with age as a result of the end replication problem, which indicates that during cell division the ends of linear DNA cannot be replicated completely during lagging strand DNA synthesis [2]. Furthermore, oxidative stress is associated with telomere shortening and dysfunction [2]. In humans, telomere length (TL) can vary from 3 to 20 kilobase pairs (kb) [3]. Cells are able to maintain TL with telomerase, a ribonucleoprotein enzyme complex that synthesizes telomeric DNA at the 3′ end of linear chromosomes [4]. It is composed of the enzymatic reverse transcriptase protein TERT (telomeric reverse transcriptase) and the RNA template TERC (telomeric RNA component) [4]. Early in human development, telomerase is constitutively active in all body cells. However, after birth it is active predominately in germline cells, activated lymphocytes, and certain types of stem cell populations, with most somatic tissues having no telomerase activity [5]. Cancer cells, on the other hand, can restore their telomeres through two known pathways, either through telomerase that is widely expressed in malignant cells, or with alternative lengthening of telomeres (ALT) [6]. The alternative lengthening pathway is rarer and based on the elongation of telomeres upon recombination. Thus, it is characterized by continuous cell growth in the absence of telomerase [7].

Telomere length is highly variable in adults, as well as in children [8]. After birth, TL starts to decrease rapidly, indicating that during pregnancy TL is maintained with specific to life in utero mechanisms [9,10,11]. It is important to note that in adulthood, gender influences TL with women demonstrating longer TL than men [12,13,14]. Furthermore, although TL is highly variable in somatic tissues of adults, it has been shown that telomeres shorten at equivalent rates [15,16]. The rate of telomere shortening is high during the first three years of life (approximately 250 bp/year) and reaches a plateau at the age of four years. From the age of four years onwards and till young adulthood, this rate of telomere attrition is either stable or declines (up to 50 bp/year), and reduces further in older individuals (approximately 50 bp/year) [17,18,19,20,21]. Different periods of childhood are characterized by differences in telomere attrition rates owing to an increased cell replication rate in infants, especially during the accelerated development of the immune system, while in adults with obesity, telomere attrition rate was greater in those with higher TL prior to a lifestyle intervention [13,22,23]. In the early stages of life and adulthood, short TL or high rates of telomere attrition have been associated with lower life expectancy and higher disease risk in humans, as well as in other species [24,25,26,27,28,29,30,31]. As previously stated, during embryonic development, the telomerase is active in the embryo, enabling the maintenance of equally long telomeres in all tissue cells and causing cells of different tissues to have similar TL [32,33,34]. With ageing, different tissue cells lose the similarity in TL mainly due to the respective differences in their proliferative activity [16]. However, similar rates of age-dependent attrition have been reported in leucocytes, skin, fat, and muscle cells [16]. A child or an adult with long leukocyte telomere length (LTL) will have long muscle telomere length (MTL), indicative of the existence of a synchrony across these somatic tissues in the same individual [35].

Telomere length is a highly heritable trait (36–86% in family and twin studies) [36,37,38,39,40,41] and in newborns, it appears to be associated with parental TL [41,42,43,44]. Apart from genetic factors, TL can be altered by factors, such as gender (women have longer TL than men) [45], race (African Americans have longer TL than Europeans) [46,47], paternal age (older age of the father is associated with longer TL) [48,49], smoking (smokers have shorter TL than non-smokers) [50,51], physical activity (non-active subjects have shorter TL than active subjects) [52], traumatic events (cause shortening of TL) [53,54,55,56], obesity (causes shortening of TL) [51], and oxidative stress (causes shortening of TL) [57,58]. Moreover, specific conditions may accelerate TL attrition even in children via oxidative, immune, hormonal, and metabolic pathways, linking these pathophysiological mechanisms and TL with the development or susceptibility to disease [59,60].

The prevalence of obesity has been increasing rapidly worldwide during the last four decades, with a concomitant rise in the prevalence of obesity in childhood and adolescence [61]. Obesity is characterized by “abnormal” or excessive body fat accumulation, with complications that include insulin resistance, glucose intolerance, diabetes mellitus type 2, dyslipidemia, hypertension, early onset atherosclerotic cardiovascular disease, endothelial dysfunction, orthopedic problems, hypogonadism, social stigmatization, fatty liver disease, cholecystitis, and increased incidence of certain malignancies [62,63,64,65,66,67,68]. Overweight and obesity in childhood results in obesity in adulthood and is associated with significant morbidity and mortality early in adult life [69]. The recognition of adipose tissue as an endocrine organ capable of secreting adipokines, which facilitate communication between adipose tissue and other organs and influence whole-body energy homeostasis, increased our understanding on the pathophysiology of obesity [70]. Adipokines have pro- and anti-inflammatory actions that might contribute to the onset and progression of obesity-related disorders [71]. Obesity in childhood is also associated with chronic inflammation, which is mediated by cytokines (and adipocytokines), increased reactive oxygen and nitrogen species (RONS) production (pro-oxidation, decreased anti-oxidation), and increased systemic oxidative stress [51,72,73,74,75]. 

Furthermore, obesity may accelerate telomere attrition rates and biological ageing, and BMI in adulthood is inversely associated with TL, an important fact given that the increase of telomere attrition and reduced telomere length in various age-related diseases is associated with increased morbidity and mortality [76,77]. Increased BMI results in higher blood volume, increased proliferation of blood cells, and telomere shortening, whereas weight loss has been positively correlated with telomere lengthening [51]. As previously noted, obesity is mechanistically associated with chronic, low-grade inflammation and increased oxidative stress [69,72]. At the same time chronic inflammation and oxidative stress, even in the absence of obesity (e.g., aging associated morbidity), are associated with increased telomere attrition and shorter TL [78,79,80]. The strong association of obesity with low-grade inflammation and oxidative stress underlines the importance of the study of the impact of telomere attrition during childhood on later life, particularly given that there is an inverse correlation between TL and BMI, total fat, and waist circumference, and that abdominal adipose tissue is directly related to TL attrition and promotion of the aging process [81]. It is important to note that adequate treatment of obesity in childhood potentially prevents complications of obesity in adulthood [82]. In addition, individuals who have shorter TL before intervention benefit from the most pronounced rate of telomere lengthening after weight loss [74], while better compliance to a Mediterranean diet is also associated with longer TL [51,72,73,74].

In order to systematically summarize current knowledge on the association between childhood obesity and TL, we undertook the present systematic review. The primary objective of the present systematic review was to summarize the current knowledge on the effects of childhood obesity (<18 years) on TL. The secondary objective was to assess the effect of weight management interventions on TL in childhood obesity (<18 years).

## 2. Materials and Methods

### 2.1. Study Design

The present study was conducted according to the Preferred Reporting Items for Systematic Reviews (PRISMA) protocol [83]. The systematic review was registered in the OSF registries with Registration DOI 10.17605/OSF.IO/Q5BTG. The study protocol included the following consecutive stages: (i) primary research of the systematic literature using search engines; and (ii) selection of studies to be included according to our inclusion and exclusion criteria.

### 2.2. Eligibility Criteria

The eligibility criteria were predetermined by the authors (Table 1). We included all original studies that assessed the relation between TL and obesity, overweight or normal-BMI in children and adolescents aged <18 years old. In some studies, the population included both adults and children; we evaluated only those that assessed children separately. In order to prevent language bias, our search was not limited by language. There was no date or country restriction. Reviews were excluded from our study selection. 

### 2.3. Literature Search

Two independent reviewers used the PubMed, Scopus, Web of Science and www.Heal-link.gr (accessed on 20 January 2022) search engines in primary search prior to September 2021. 

Our search strategy for the primary objective included the terms: “telomer*” [All Fields] AND (“length” [All Fields] OR “lengths” [All Fields]) AND “obes*” [All Fields] AND (“child*” [All Fields] OR “adolescen*” [All Fields]). 

A second search with the search terms for weight management interventions (secondary objective search) was also performed: “telomer*” [All Fields] AND (“length” [All Fields] OR “lengths” [All Fields]) AND “obes*” [All Fields] AND (“child*” [All Fields] OR “adolescen*” [All Fields]) AND ((“weight s” [All Fields] OR “weighted” [All Fields] OR “weighting” [All Fields] OR “weightings” [All Fields] OR “weights and measures” [MeSH Terms] OR (“weights” [All Fields] AND “measures” [All Fields]) OR “weights and measures” [All Fields] OR “weight” [All Fields] OR “body weight” [MeSH Terms] OR (“body” [All Fields] AND “weight” [All Fields]) OR “body weight” [All Fields] OR “weights” [All Fields]) AND (“manage” [All Fields] OR “managed” [All Fields] OR “managements” [All Fields] OR “managements” [All Fields] OR “manager” [All Fields] OR “manager s” [All Fields] OR “managers” [All Fields] OR “manages” [All Fields] OR “managing” [All Fields] OR “managment” [All Fields] OR “organization and administration” [MeSH Terms] OR (“organization” [All Fields] AND “administration” [All Fields]) OR “organization and administration” [All Fields] OR “management” [All Fields] OR “disease management” [MeSH Terms] OR (“disease” [All Fields] AND “management” [All Fields]) OR “disease management” [All Fields])). Articles together with reference lists from included studies were retrieved.

### 2.4. Study Selection

After the initial search, all obtained documents were screened independently by the two reviewers. The two reviewers used a standard Excel extraction datasheet to exclude all duplicates and examined eligibility for the studies based on the relevance of their title and abstract. For the relevant articles, full text was assessed based on the inclusion and exclusion criteria. All included studied were of ‘very good quality’ (9–10 points: 4 cross-sectional and 2 intervention) and ‘good quality’ (7–8 points: 7 cross sectional and 3 intervention). Therefore, the overall risk of bias was not high (Table 2). During the whole procedure, each reviewer remained blinded to the other investigator’s selection and finally, after consensus, 16 articles were used in the qualitative analysis of this review.

## 3. Results

### 3.1. Literature Search

**Figure 1:**
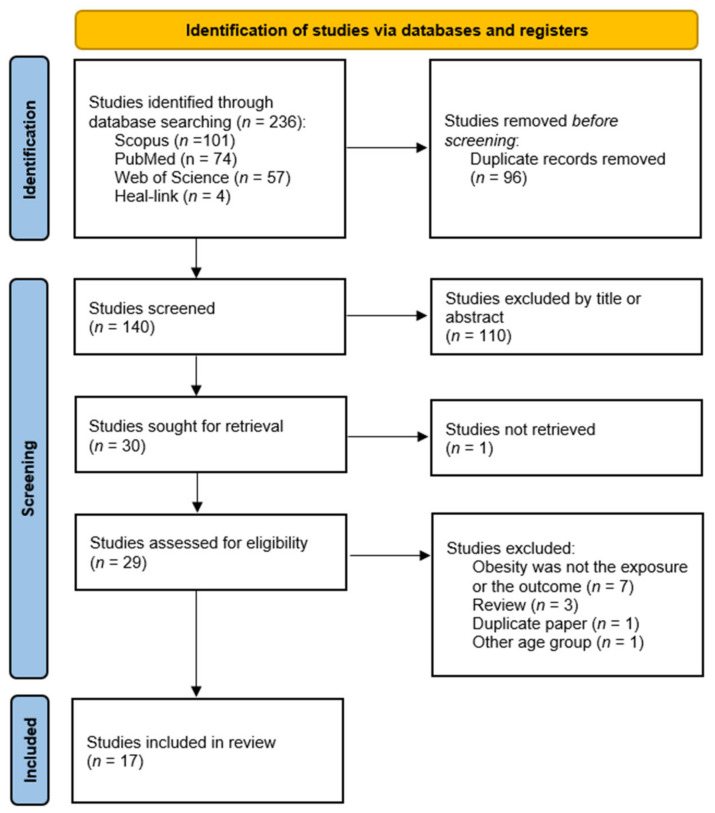
Flow diagram of the study selection process.

A total of 236 articles were identified by two independent researchers using the search terms listed above. After removing 96 duplicates, 110 irrelevant papers were excluded after the title and abstract screening. A total of 29 papers were eligible for full-text review. Among these full-text articles, 13 were excluded for the following reasons: namely reviews/systematic reviews, duplicate papers, other age group or information that did not meet the eligibility criteria and the purpose of this systematic review. Finally, 16 full-text articles [84,85,86,87,88,89,90,91,92,93,94,95,96,97,98,99] were considered eligible and were included to our qualitative synthesis. The study selection procedure is detailed in the respective flow chart (Figure 1).

### 3.2. Study Characteristics 

In the systematic review for the primary objective, ten cross-sectional studies [84,85,86,87,88,89,90,91,93,94] and one case control [92] were included (Table 3). The age ranged from 2 to 18 years and the percentage of boys was between 46.0% and 67.7%. The majority of the included studies (15/16) used quantitative real-time PCR (qPCR) to evaluate telomere length [84,85,86,87,88,89,90,91,92,93,95,96,97,98,99], while one study (1/16) used terminal restriction fragmentation (TRF) method [94]. Most studies (8/11) measured telomere length from leukocytes. 

As for the obesity definition, only one study divided subjects in three groups (obese, overweight and of normal-BMI) according to the IOTF criteria [89]. When the overweight group was not included, three studies compared obese and normal-BMI children according to the IOTF and WHO criteria [74,85,86,92]. In five studies, the overweight and obese children were grouped together, according to the CDC and WHO criteria [84,87,88,90,91]. Of the included studies, five showed significantly shorter TL in overweight and obese children compared to children with normal-BMI [85,86,88,89,92], while five did not find any significant differences [84,87,90,91,94]. One study revealed shorter TL only in obese boys compared with their normal-BMI counterparts, but not in girls [93]. 

In the systematic review for the secondary objective, we included five intervention studies shown in Table 4. The ages ranged from 7 to 16 years and the percentage of boys was between 36.0% and 48.6%. All of the included studies used quantitative real-time PCR (qPCR) to evaluate telomere length and measured telomere length from leukocytes [95,96,97,98,99]. Lifestyle interventions in all five studies included dietary and physical activity interventions, and in all of them, participants are reported to have lost weight. Of the five studies that evaluated the effect of a lifestyle intervention program in obese or overweight children, three demonstrated a significant increase in TL as a result of a decrease in BMI [95,98,99]. In two of the studies, TL increased following two months of intervention, while in the study conducted by our group, TL increased in all subjects following twelve months of intervention [95]. In two studies, TL did not show a significant change [96,97]. Both studies had an intervention period of two months that may be too short a time (depending on the intervention) to result in TL increase [96,97].

## 4. Discussion

Among the studies assessed in this systematic review, there was only one study that showed a significantly greater TL in normal-BMI than in overweight and obese children. In this study, there was no difference in TL between the overweight and obese groups [89]. When the overweight group was not included, three studies showed significantly shorter TL in obese than in normal-BMI children [85,86,92,93]. In four studies, no difference in TL between normal-BMI and the combined overweight/obese children was found [84,87,90,91], while in one study TL was greater in normal-BMI children [88]. It is important to note that, in the four studies that showed no difference in TL among groups, the CDC criteria were used for the definition of overweight and obesity, while the fifth one used the WHO criteria for the definition of overweight and obesity. The growth charts designed by WHO are more sensitive in identifying obesity in a population at risk, which has important implications for the prevention and management of the condition [100]. It has been shown that the WHO growth charts yield a higher prevalence estimate of being overweight and obesity since the reference population is intended to be a non-obese sample, whereas CDC and IOTF are derived using more recent data in which the BMI distribution of the reference populations is already shifted toward the right due to the recent increase in the BMI in childhood and adolescence [101]. Thus, more children with obesity are classified as having a normal BMI via the CDC and IOTF criteria, and this limitation should be taken into account when making comparisons between the two groups. 

When assessing the correlation between BMI and TL in two of the included studies, a statistically significant negative correlation was found [85,88]. In the other two included studies that assessed the respective correlations, no association was reported [86,92]. It has been shown previously that TL correlates with anthropometric outcomes, such as BMI in adults, with the majority of studies reporting an inverse relation between BMI and TL in adulthood [102,103,104]. However, according to the systematic review and meta-analysis of Guyatt et al., there was no strong evidence for an association between any adiposity measure in neonates and children and TL [105]. 

The exact mechanisms underlying the association between measures of obesity and short TL still needs to be elucidated. Increased oxidative stress and the persistent activation of inflammatory processes [72,106] are the key factors that may explain the association between telomere attrition and obesity, as well as cardiometabolic disorders in adulthood [107,108,109,110,111]. Oxidative stress is a state of imbalance between pro- and anti- oxidation within the cell [112,113]. Pro-oxidation refers to mitochondrial and non-mitochondrial mechanisms, which generate reactive oxygen and nitrogen species [112,113]. Antioxidation is the adaptive activation of enzymatic and nonenzymatic mechanisms that counterbalance pro-oxidation by activating the scavengers of pro-oxidants and their products within cells and in extracellular body fluids [112,113,114]. Obesity and its comorbidities in humans are associated with increased pro- and decreased anti-oxidation, respectively, even in childhood [72,113,115,116]. Increased levels of oxidative stress are considered detrimental to cell signal transduction [117], potentially explaining the reported correlation between increased oxidative stress and shorter TL [118,119,120]. The adipose tissue plays a role in the production of inflammatory cytokines and chemokines, as well as in host defense, immunity, and injury response [121]. Furthermore, adipose tissue-related aseptic low-grade inflammation might contribute to increased production of reactive oxygen and nitrogen species (RONS) and reduced antioxidant capacity [72,75,122,123,124,125]. Another proposed mechanism links the proinflammatory state that accompanies obesity with hyperplasia and hypertrophy of adipocytes, which can induce adipose tissue hypoxia [126]. This adipose tissue dysfunction causes secretion of prostaglandins, C-reactive protein, and proinflammatory cytokines, such as interleukin-6, tumor necrosis factor, and adipokines, such as leptin [69,127]. It also lowers the concentrations of anti-inflammatory adipokines, such as adiponectin, even in childhood [75]. The increased BMI in childhood and adolescence may be associated with accelerated biological ageing and may have an adverse impact on future health in adulthood, such as cardiometabolic diseases [79,89,128,129]. Obesity is regarded as a crucial factor in the regulation of adipose tissue aging and further metabolic outcomes, such as increased proinflammatory cytokines, insulin resistance, diabetes mellitus type 2, and cardiovascular disease [79,128,129]. The p53 pathway in adipose tissue, which is a key factor in the aging process of adipose tissue and increased inflammation, may play an important role in relation to obesity and obesity-mediated aging [129,130]. These DNA damage responses are defined by the consequences of genomic instability, a reaction of cells to damaged DNA to prevent negative health conditions, such as the initiation of mitotic senescence, arrest, repair, and cell death [131,132,133].

Although BMI is an important indicator of adiposity, studies examining the impact of specific markers of adiposity have shown that there is a negative correlation between TL and waist circumference [86,88,93], as well as between TL and skinfold thickness [88] (although Ooi et al. found a positive correlation between TL and skinfold measurements) [86]. Studies in adults showed a negative correlation between adiposity markers and TL [76]. It is important to note that several previous studies have suggested that the relationship between TL and adiposity is stronger in younger adults than in older adults [13,104,134,135]. This has been attributed to the accumulated damage due to inflammatory processes and oxidative stress over the life course, which may be greater in the elderly [104]. Indeed, increased adipose tissue markers, such as increased waist circumference and adipose tissue, as assessed by skinfold thickness, have been associated with markers of cardiovascular risk, chronic inflammatory state and oxidative stress. Therefore, they could be associated with shorter TL [72]. Inflammatory process-induced DNA damage may explain the association between obesity and telomere shortening [110]. Previous studies have shown a significant inverse relation between the levels of inflammation and TL in both adults [78] and children [136]. In obesity, increased adipose tissue results in increased production of pro-inflammatory adipokines and cytokines [137] and potentially TL shortening [106,119]. 

Of the five studies that evaluated the effect of a lifestyle intervention program in obese or overweight children, three demonstrated a significant increase in TL as a result of a decrease in BMI [95,98,99]. Two studies had an intervention period of two months that may be too short a time (depending on the intervention) to result in TL increase [96,97]. In addition, in both studies the sample size was small (≈100) [96,97]. Physical activity and exercise are important interventions that support and accelerate the process of weight loss in addition to an effective diet plan. They have protective and restorative effects and, therefore, improve well-being and increase longevity. Physical activity stimulates the metabolism, including oxidative, inflammatory, and neuroendocrinological adaptive systems [80]. Several exercise-only interventions have demonstrated a reduction in the biomarkers of oxidative stress [80,138,139,140,141]. Little is known about the differences of oxidative stress-related processes between obese and non-obese individuals [116,142]. In addition to physical activity, weight loss itself may reverse telomere attrition. Increased consumption of healthy foods typical of the traditional Mediterranean diet (vegetables, fruits, nuts, wine, and coffee, foods rich in antioxidants, and other phytochemicals) has been consistently associated with telomere lengthening, whereas increased consumption of processed meat and sweetened beverages has quite the opposite effect [143]. Also, intake of foods rich in antioxidants leads to healthy cell aging, which can delay telomeric friction [144]. The consumption of foods rich in antioxidants and anti-inflammatory compounds plant-based foods has beneficial effects on several anti-inflammatory markers and in reducing oxidative stress and cardiovascular risk factors [145,146].

Telomere length from peripheral white blood cells correlates very well with their bone marrow residing precursors, justifying the quantification of TL from peripheral blood cells as an indicator of stem cell divisions [147]. Telomere lengthening could be explained by mechanisms involved in either the reduction of biological stress (inflammation or oxidative stress) [148,149] or the redistribution in circulating immune cells (naïve T cells) regardless of cell type (including diverse populations), such as large numbers of younger cells with longer telomeres [150,151]. Within redistribution, there can be increases in numbers of specific types of naive cells regardless of cell type. Biegler and colleagues’ findings suggest that lengthening was associated with an influx of naive T cells [152]. Naive T cells naturally have longer TL than memory T cells, but may also have longer TL than granulocytes [19]. Any factors that stimulate greater naive T-cell influx may lead to lengthening of average TL (mimicking the reversal typically associated with telomerase-mediated per cell increases in base pairs). Thus, rather than elongation of TL on a per cell basis, it seemed to be in part due to replenishment of cells in circulation. Younger cells in the circulation were shown to positively influence longevity in experimental animals via an unknown mechanism [153]. This observation may lead to future studies examining the potential mechanisms with which lifestyle intervention/weight management can lead to redistribution of younger cells in the body. 

Improvements in health behaviors might promote short-term lengthening. Exercise may be one of the important factors shaping apparent or actual lengthening, as it is related to greater numbers of naive cells, as well as longer TL [154,155]. Improving well-being through decreasing stress and concomitant increases in growth and restorative factors might induce a specific pattern of adhesion molecule-stimulated trafficking patterns and result in cells with longer telomeres, either more naive cells or fewer senescent cells. Accordingly, age-dependent decline in TL of mononuclear bone marrow cells was similar to peripheral blood cells, suggesting an ageing process already in stem and progenitor cells, indicating that changes in the periphery might reflect changes in hematopoiesis [147].

## 5. Limitations

The main limitation of our study was the variations in study size and age structure of the study population that can both contribute towards the heterogeneity of the results [96,97]. In addition, the different approaches in obesity definition may also contribute to the heterogeneity of the results. In addition, the heterogeneity between populations should be considered, given that TL is influenced by both genetic factors and ethnicity [46,84]. Furthermore, the TRF assay provides a readily quantifiable distribution of TLs in kb units, which can be compared across populations and species and used to estimate both mean, medians, and variance in TLs within a sample of cells [156]. On the other hand, qPCR provides only average TL as a relative ratio. Comparing the results of the two techniques will provide limited comparability. It is important to note that the study that compared TL using the TRF technique in normal-BMI and obese children, showed no difference in TL between the two groups [94].

## 6. Conclusions

The results of this systematic review are consistent with the proposed biological mechanism that increased adiposity leads to telomere shortening, which can hasten the ageing process even in childhood. However, the available data are still sparse and some individual studies report contradicting associations that may be inherent to the variability in study design (and particularly the definition of obesity). 

Furthermore, increasing evidence supports the hypothesis that the beneficial effects of lifestyle intervention processes can be associated with increased TL in the periphery. The apparent reversal of the well-documented age-dependent decline in measured telomere length from peripheral leukocytes reflects changes in hematopoiesis and the possible redistribution of younger cells in the periphery, indicating a potential contributing mechanistic factor to these beneficial effects.

## Figures and Tables

**Table 1:** Inclusion and exclusion criteria.

Inclusion Criteria		Exclusion Criteria
Age < 18 years old Studies from any geographic location Any publication date Any language Assessing the relationship between telomere length and obese, overweight and normal-BMI children and adolescents		Age ≥ 18 years old Reviews, editorials, abstracts, case controls, expert opinions In vitro or animal experiments
Primary objective Observational studies	Secondary objective Interventional studies	

**Table 2:** Ottawa scale.

**Primary Objective: Cross-Sectional Studies**				
**Author/Year**	**Selection**	**Comparability**	**Outcome**	**Overall Score**
Selvaraju 2021 [84]	** **	**	***	9
Liu 2021 [85]	** **	*	***	8
Ooi 2021 [86]	** **	*	***	8
Licea-Cejudo 2020 [87]	** **	*	***	8
Clemente 2019 [88]	** **	**	***	9
Lamprokostopoulou 2019 [89]	*****	*	***	9
Theall 2019 [90]	** **	*	***	8
Zhu 2015 [91]	** **	**	***	9
Buxton 2011 [92]	** **	*	***	8
Al-Attas 2010 [93]	** **	*	***	8
Zannolli 2008 [94]	* **	*	***	7
**Secondary Objective: Interventional Studies**				
**Author/Year**	**Selection**	**Comparability**	**Outcome**	**Overall Score**
Paltoglou 2021 [95]	** **	*	***	8
Ojeda-Rodríguez 2021 [96]	*****	*	***	9
Morell-Azanza 2020 [97]	** **	*	***	8
Ojeda-Rodríguez 2020 [98]	** **	*	***	8
García-Calzón 2014 [99]	*****	*	***	9

The Newcastle-Ottawa Scale quality instrument is scored by awarding a point for each answer that is marked with an asterisk (*). Each asterisk represents the fact that an individual criterion within the subsection was fulfilled.

**Table 3:** Characteristics of the cross-sectional studies included in the systematic review.

Author Year	Nationality	Study Design	Sample Size		Obesity Definition	Age (Years) Mean ± SD or Range	Male (%)	Method/Tissue	TL Outcome
			Total	OB & OW (%)	Reference Grouping				
Selvaraju 2021 [84]	European American, African American	Cross-sectional	127	NA	CDC [NW]-([OW] & [OB])	6–10	NA	qPCR Saliva	▪ No differences in the TL among obese, overweight and normal-BMI groups of either race
Liu 2021 [85]	Chinese	Cross-sectional	92	50% OB	WHO [NW]-[OB]	3–4	NA	qPCR Leukocytes	▪ Significantly shorter TL in obese children compared to children with normal BMI ▪ Negative correlation between TL and BMI
Ooi 2021 [86]	Singaporean	Cross-sectional	394	94.2% OB	WHO + 2SD [NW]-[OB]	14.0 ± 3.02 OB 13.1 ± 3.40 NW	67.7	qPCR Leukocytes	▪ Significantly shorter TL in obese children compared to children with normal BMI ▪ Negative correlation between TL and waist circumference ▪ Positive correlation between TL and skinfold measurements
Licea-Cejudo 2020 [87]	Mexican	Cross-sectional	134	65.7% OB	CDC [NW]-([OW] & [OB])	8–10	52	qPCR Saliva	▪ No differences in the TL among obese, overweight and normal-BMI children ▪ Significantly shorter TL were associated with high BF% in boys, but not in girls
Clemente 2019 [88]	European	Cross-sectional	1.396	6% OB 15.4% OW	WHO [NW]-([OW]&[OB])	6–11 8.0 ± 1.5	53.9	qPCR Leukocytes	▪ Significantly shorter TL in overweight and obese children compared to children with normal BMI
Lamprokostopoulou 2019 [89]	Greek	Cross-sectional	919	13.5% OB 30.0% OW	Greek Ref. Chart IOTF [NW]-[OW]-[OB]	9–13	50.2	qPCR Leukocytes	▪ Significantly shorter TL in overweight and obese children and adolescents compared to children with normal BMI ▪ Multivariable linear regression revealed increasing weight category was inversely associated with TL in children and adolescents
Theall 2019 [90]	American	Cross-sectional	90	32.2% OW&OB	CDC [NW]-([OW] & [OB])	5–16	46%	qPCR Buccal swab	▪ No differences in the TL among obese, overweight and normal-BMI children
Zhu 2015 [91]	Caucasian and Afro-American	Cross-sectional	766	24.9% OW&OB	CDC [NW]-([OW] & [OB])	14–18 16.1 ± 0.0	49.7	qPCR Leukocytes	▪ No differences in the TL among obese, overweight and normal-BMI children ▪ Significantly shorter TL in overweight and obese adolescents is associated with higher dietary sodium intake
Buxton 2011 [92]	French	Case-control	793	59.4% OB	French Ref. Chart [NW]-[OB]	2–17	53.9	qPCR Leukocytes	▪ Significantly shorter TL in obese girls and boys compared with their non-obese counterparts
Al-Attas 2010 [93]	Saudi Arabian	Cross-sectional	148	35.1% OB	IOTF [NW]-[OB]	5–12 8.5 ± 2.1 boys 9.7 ± 2.6 girls	46.6	qPCR Leukocytes	▪ Significantly shorter TL in obese boys compared with their normal-BMI counterparts, but not in girls
Zannolli 2008 [94]	Italian	Cross-sectional	53	22.6% OB	Italian Ref. Chart [NW]-[OB]	8.2 ± 3.5	NA	TRF PBMC	▪ No differences in the TL between obese and normal-BMI children

Abbreviations: NA, not available; TL, telomere length; OW, overweight; OB, obese; NW, normal weight; non obese, normal (BMI) weight and overweight; CDC: Center of Diseases Control and Prevention; WHO, World Health Organization; IOTF, International Obesity Task Force; PCR, polymerase chain reaction.

**Table 4:** Characteristics of the lifestyle intervention studies included in the systematic review.

Author Year	Nationality	Dietary and Physical Activity Interventions		Sample Size		Obesity Definition	Age (Years) Mean ± SD or Range	Male (%)	Method/ Tissue	TL Outcome
		Period	Telomere Measure	Total	OB & OW (%)	Reference Grouping				
Paltoglou 2021 [95]	Greek	12 months	Baseline 12 months	508	52.6% OB 34.2% OW	Greek Ref. Chart [NW][OW] [OB]	10.14 ± 0.13	47	qPCR Leukocytes	▪ A significant increase in TL irrespective of gender, pubertal status, or BMI after a lifestyle intervention program of healthy diet and physical exercise
Ojeda-Rodríguez 2021 [96]	Spanish	2 months	Baseline 2, 12 months	102 OW&OB	75 Intervent. 27 usual care	Spanish Ref. Chart [OW&OB]	7–16	36	qPCR Leukocytes	▪ TL did not change after intervention ▪ TL increase was positively associated with energy expenditure as assessed by metabolic equivalents (METs), moderate physical activity (MVPA) level and number of steps in the intervention group after the intensive phase ▪ TL increase was inversely associated with secondary and light physical activity (PA) levels in the intervention group after the intensive phase
Morell-Azanza 2020 [97]	Spanish	2 months	Baseline 2 months	106 OW&OB		Spanish Ref. Chart [OW&OB]	7–16 11.30 ± 2.49	37	qPCR Leukocytes	▪ TL did not change after intervention ▪ A negative TL correlation with BMI at baseline ▪ TL at baseline predicted changes in blood glucose levels after the intervention
Ojeda-Rodríguez 2020 [98]	Spanish	2 months	Baseline 2, 12 months	87 OW&OB	64 intervent. 23 usual care	Spanish Ref. Chart [OW&OB]	7–16	39	qPCR Leukocytes	▪ A significant increase in TL in the follow up period 2–12 months of intervention, in children with abdominal obesity enrolled in an intensive lifestyle intervention
García-Calzón 2014 [99]	Spanish	2 months	Baseline 2 months Follow-up 6 months	74 OW&OB	96% OB 4% OW	Spanish Ref. Chart [OW&OB]	12–16	48.6	qPCR Leukocytes	▪ A significant increase in TL in overweight & obese adolescents after a weight loss intervention
